# Free Fatty Acids, Lipopolysaccharide and IL-1α Induce Adipocyte Manganese Superoxide Dismutase Which Is Increased in Visceral Adipose Tissues of Obese Rodents

**DOI:** 10.1371/journal.pone.0086866

**Published:** 2014-01-24

**Authors:** Sabrina Krautbauer, Kristina Eisinger, Markus Neumeier, Yvonne Hader, Roland Buettner, Peter M. Schmid, Charalampos Aslanidis, Christa Buechler

**Affiliations:** 1 Department of Internal Medicine I, Regensburg University Hospital, Regensburg, Germany; 2 Department of Internal Medicine II, Regensburg University Hospital, Regensburg, Germany; 3 Institute of Clinical Chemistry and Laboratory Medicine, Regensburg University Hospital, Regensburg, Germany; Universidad Pablo de Olavide, Centro Andaluz de Biología del Desarrollo-CSIC, Spain

## Abstract

Excess fat storage in adipocytes is associated with increased generation of reactive oxygen species (ROS) and impaired activity of antioxidant mechanisms. Manganese superoxide dismutase (MnSOD) is a mitochondrial enzyme involved in detoxification of ROS, and objective of the current study is to analyze expression and regulation of MnSOD in obesity. MnSOD is increased in visceral but not subcutaneous fat depots of rodents kept on high fat diets (HFD) and ob/ob mice. MnSOD is elevated in visceral adipocytes of fat fed mice and exposure of differentiating 3T3-L1 cells to lipopolysaccharide, IL-1α, saturated, monounsaturated and polyunsaturated free fatty acids (FFA) upregulates its level. FFA do not alter cytochrome oxidase 4 arguing against overall induction of mitochondrial enzymes. Upregulation of MnSOD in fat loaded cells is not mediated by IL-6, TNF or sterol regulatory element binding protein 2 which are induced in these cells. MnSOD is similarly abundant in perirenal fat of Zucker diabetic rats and non-diabetic animals with similar body weight and glucose has no effect on MnSOD in 3T3-L1 cells. To evaluate whether MnSOD affects adipocyte fat storage, MnSOD was knocked-down in adipocytes for the last three days of differentiation and in mature adipocytes. Knock-down of MnSOD does neither alter lipid storage nor viability of these cells. Heme oxygenase-1 which is induced upon oxidative stress is not altered while antioxidative capacity of the cells is modestly reduced. Current data show that inflammation and excess triglyceride storage raise adipocyte MnSOD which is induced in epididymal adipocytes in obesity.

## Introduction

Adipocytes control whole body energy homeostasis through the storage of triglycerides and release of fatty acids during fasting [1], [2]. Adipogenesis is a complex process where preadipocytes acquire the ability to deposit lipids in lipid droplets [3]. Fatty acids are stored in the form of triglycerides and for esterification glycerol-3-phosphate and acetyl-CoA are used as substrates. Synthesis of these metabolites depends on mitochondrial function and adipogenesis is accompanied by mitochondrial biogenesis [4], [5]. Mitochondria metabolize oxygen and are a major source of reactive oxygen species (ROS) [6]. During adipogenesis of 3T3-L1 adipocytes expression of manganese superoxide dismutase (MnSOD), Cu/Zn SOD and catalase are induced [7]. Generation of superoxide is increased in mature adipocytes and higher expression of these enzymes may help to balance cellular ROS [4], [7].

In obesity high levels of free fatty acids (FFA) contribute to inflammation and oxidative stress, and adipocytes exposed to excess FFA produce ROS [8]–[10]. Saturated and unsaturated FFA have been shown to increase ROS in 3T3-L1 cells after 24 h of incubation [11]–[13]. Differentiation of these cells in medium with palmitate also enhances ROS production while ROS are not induced by stearate, oleate and linoleate [10]. These discordant findings may be partly explained by the different FFA concentrations and incubation times examined. Furthermore, exposure of already differentiated adipocytes to FFA may have other effects than differentiation of these cells in the presence of FFA [10]–[13].

Higher production of ROS in FFA incubated adipocytes is explained by mitochondrial dysfunction, increased activity of NADPH oxidase and lower antioxidative capacity [11], [12], [14], [15]. Palmitate reduces glutathione peroxidase and increases glutathione levels in 3T3-L1 adipocytes and stearate lowers MnSOD mRNA in these cells [12], [15]. Antioxidant capacity of adipose tissue is also impaired in animal models of obesity, and antioxidants like SOD mimetics exert beneficial effects in metabolic diseases associated with obesity [12], [15]–[17].

Mitochondrial content and expression of mitochondrial genes are markedly reduced in obesity [5], [18]–[20]. Lower mitochondrial activity is found in epididymal adipose tissues [5], [18], [20] and Rong et al describe reduced mitochondrial biogenesis in subcutaneous fat depots [19]. Impaired mitochondrial activity is suggested to increase ROS which contribute to inflammation and insulin resistance [19]–[21]. Several studies have, however, shown that mitochondrial dysfunction may even protect from obesity and insulin resistance indicating that reduced mitochondrial activity may be a consequence rather than a cause of obesity [22]–[24]. Although the role of ROS in metabolic diseases associated with obesity is still unclear, ROS are clearly increased whereas antioxidant activity is decreased [8], [25]. One enzyme for scavenging ROS is MnSOD, a nuclear encoded mitochondrial gene. MnSOD deficient mice die within the first 10 days of life demonstrating the physiological importance of this protein [26]. In heterozygous MnSOD knockout mice MnSOD protein is reduced by about 70% in muscle and fat, and glucose tolerance is already impaired when these mice are fed a standard chow [9].

Here, MnSOD was determined in adipose tissues of rodents kept on high fat diets, Zucker diabetic rats and ob/ob mice. Regulation of MnSOD by FFA, IL-1α, glucose and LPS was analyzed in 3T3-L1 cells. The function of this protein in adipocyte triglyceride storage was studied in 3T3-L1 cells using RNA Interference techniques.

## Materials and Methods

### Culture Media and Reagents

MnSOD antibody was from Thermo Fisher Scientific (Schwerte, Germany). Antibodies to β-actin, cytochrome C oxidase-4 (Cox-4), cyclophilin A, GAPDH, fatty acid binding protein 4 (FABP4), poly ADP ribose polymerase (PARP) and steaoryl CoA desaturase 1 (SCD1) were from New England Biolabs GmbH (Frankfurt, Germany). Heme oxygenase 1 antibody was from Novus Biologicals (Cambridge, UK). Monoclonal anti CD163 antibody was from Morphosys AbD (Matinsried, Germany). Palmitate (PA), oleate (OA), linoleate (LA) and lipopolysaccharide (LPS) were ordered from Sigma (Deisenhofen, Germany). OxiSelect™ Oxygen Radical Antioxidant Capacity (ORAC) Activity Assay was from Cell Biolabs Inc (San Diego, CA, USA). IL-6, TNF and IL-1α were measured by ELISA (R&D Systems, Wiesbaden-Nordenstadt, Germany) as partly described [27]. Recombinant TNF and IL-1α were from R&D Systems and IL-6 was from Miltenyi Biotech GmbH (Bergisch-Gladbach, Germany).

### Knockdown of MnSOD and SREBP2

MnSOD small interfering RNAs (siRNAs) and Silencer Negative Control siRNA were from Applied Biosystems (Darmstadt, Germany). MnSOD siRNAs (5′ GCU CUA AUC AGG ACC CAU Utt 3′ and 5′ AGG GAG AUG UUA CAA CUC Att 3′) were used and knocked down MnSOD with similar efficiencies. The 6 d predifferentiated cells were transfected using Endo-Porter (Gene Tools LLC, Philomath, Oregon, USA) according to the protocol supplied by the company. Differentiated 3T3-L1 cells were transfected using electroporation. Cells were suspended in 100 µl Amaxa Nucleofector solution (Amaxa Nucleofector Kit L, Lonza, Wuppertal, Germany) and 3 µg siRNA was added. Electroporation was performed using Nucleofector I (Lonza, Wuppertal, Germany) and the programme A33. Knockdown of SREBP2 was performed as described [27].

### Adipocyte Cell Culture

3T3-L1 preadipocytes were purchased from the American Type Culture Collection (ATCC, Manassas, VA, USA) and cultured at 37°C and 5% CO_2_ in DMEM (Biochrom, Berlin, Germany) supplemented with 10% newborn calf serum (Sigma Bioscience, Deisenhofen, Germany) and 1% penicillin/streptomycin (PAN, Aidenbach, Germany). For adipogenesis, 3T3-L1 preadipocytes were grown to confluence and differentiated into adipocytes as described [27]. Purified human preadipocytes from subcutaneous fat of three females with a BMI of 24, 28, and 38 kg/m^2^, and purified human preadipocytes from subcutaneous and visceral fat of two females with a BMI of 40 and 51 kg/m^2^ were ordered from BioCat (Heidelberg, Germany) and differentiated as suggested by the company.

### Fatty Acid Treatment

The 200 mM FFA stock solutions were prepared in ethanol by heating at 70°C and 100 µl of 200 mM FFA stock solution was added to 900 µl of a 10% fatty acid-free bovine serum albumin (BSA, Roche, Mannheim, Germany) solution to obtain a 20 mM stock solution and incubated at 55°C. The BSA-bound FFA stock solutions or equal amounts of BSA were added to the cells at day 0 and medium was changed at day 3, 6, 7 and 8.

### Rodent Adipose Tissues

Six week old, male Wistar rats were purchased from Charles River (Sulzfeld, Germany) and fed a standard rodent chow (fat content, 11% of energy) or a high-fat diet (fat content 42% of energy, HFD) based on coconut fat as described [28]. After 12 weeks the rats were killed and perirenal fat was immediately removed. The final body weight of the 4 rats fed the HFD was 554 (529–593) g. Because of the small number of animals analyzed body weight of these rats was not statistically higher compared to the 4 rats on a SD with 513 (482–541) g.

Male Zucker diabetic fatty rats (fa/fa) and male Zucker control rats (fa/−) were obtained at an age of 5 weeks from Charles River (Wilmington, MA). Animals were maintained on Purina 5008 rat chow (protein 23%, fat 6.5%, carbohydrates 58.5%, fiber 4% and ash 8%) from Charles River (Wilmington, MA) for 20 weeks. Animals were killed and perirenal fat was removed. Final body weight of the 4 Zucker diabetic rats was 349 (295–455) g and similar to the 4 control animals with 393 (378–402) g. Blood glucose was 392 (317–456) mg/dl in the diabetic and 83 (59–92) mg/dl in control animals (p = 0.029).

Seven week old male C57BL/6 mice from Charles River (Sulzfeld, Germany) were kept on a high fat diet (HFD, 7 mice) or standard chow (SD, 7 mice) for 14 weeks. Final body weight was 36.2 (31.4–44.82) g in the HFD group and 26.1 (25.4–30.7) g (p = 0.001) in the SD group. RNA was isolated and used for real-time RT-PCR analysis.

Fourteen week old male C57BL/6 mice were kept on a HFD or SD for 14 weeks. Final body weight of the 6 mice on HFD was 39.3 (32.5–41.3) g and of the 6 mice on SD was 25.8 (23.9–27.5) g (p = 0.002). Adipose tissues were used for subsequent immunoblot analysis and immunohistochemistry.

Leptin deficient male ob/ob mice (5 animals) and respective control mice (5 animals) were purchased from Charles River (Sulzfeld, Germany) at an age of 10 weeks. Three weeks later animals were killed.

Animals had free access to food and water, and were housed with 3 to 5 animals per cage. Rising concentrations of CO_2_ were used to produce loss of consciousness and was followed by *cervical dislocation.* All procedures were approved by the University of Regensburg laboratory animal committee and complied with the German Law on Animal Protection and the Institute for Laboratory Animal Research Guide for the Care and Use of Laboratory Animals, 1999.

### SDS-PAGE and Immunoblotting

The adipose tissue was solubilized in radioimmunoprecipitation assay (RIPA) lysis buffer (50 mM Tris HCl, pH 7.5, 150 mM NaCl, 1% v/v Nonidet P-40, 0.5% v/v sodium desoxycholate and 0.1% v/v SDS). 10 to 20 µg protein was separated by SDS-polyacrylamide gel electrophoresis and was transferred to PVDF membranes (Bio-Rad, Germany). Incubations with antibodies were performed in 1% BSA in TBS or PBS, 0.1% Tween overnight. Detection of the immune complexes was carried out with the ECL Western blot detection system (Amersham Pharmacia, Deisenhofen, Germany).

### Monitoring of Gene Expression by Real-time RT-PCR

The mRNA expression of murine MnSOD, F4/80 and 18S rRNA was determined by semiquantitative real-time PCR using SYBR Green (Roche, Mannheim, Germany). Total cellular RNA was isolated with TRIzol reagent from Life Technologies GmbH (Darmstadt, Germany) and 1 µg RNA was reverse transcribed using the Promega Reverse Transcription System (Promega, Madison, WI) in a volume of 40 µl; 2 µl of the cDNA was used for amplification in glass capillaries (LightCycler) using PCR primers specific for murine MnSOD (5′ GAC CCA TTG CAA GGA ACA A 3′and 5′ CAC ACA GAG TAT GCG CTG TT 3′), F4/80 (5′ TGC TCT TCC TGA TGG TGA GA 3′ and 5′ CCC CGT CTC TGT ATT CAA CC 3′) and 18S rRNA (5′ GAT TGA TAG CTC TTT CTC GAT TCC 3′and 5′ CAT CTA AGG GCA TCA CAG ACC 3′). These oligonucleotides were synthesized by Metabion (Planegg-Martinsried, Germany). Real-time RT-PCR was performed using the LightCycler FastStart DNA Master SYBR Green I kit (Roche, Mannheim, Germany) and the specificity of the PCRs was confirmed by sequencing of the amplified DNA fragments (Geneart, Regensburg, Germany). For quantification of the results RNA of adipose tissue was reverse transcribed, cDNA was serially diluted and used to create a standard curve for each of the genes analyzed. The second derivative maximum method was used for quantification with the LightCycler software. Values were normalized to 18S rRNA expression.

### Immunohistochemistry

Immunohistochemical studies for the expression of MnSOD utilized the EnVision+ Kit (DAKO, Glostrup, Denmark) based on a HRP labelled polymer which is conjugated with a secondary antibody. Five µm sections were cut from formalin-fixed and paraffin-embedded mouse adipose tissues. After deparaffinization for 20 min in Histol, tissue sections were rehydrated in descending ethanol series following antigen retrieval (microwave oven for 20 min at 800 W in sodium citrate buffer). Endogenous peroxidase activity was eliminated by subsequent incubation with peroxidase block for 10 min. After washing in TBS, 0.5% Tween slides were incubated for 1 h in a protein-blocking solution (DAKO). Incubation with the MnSOD antibody (1∶200-fold diluted) was performed overnight at 4°C in a humid chamber. After thorough washing with TBS, 0.5% Tween 20, tissue sections were incubated with anti-rabbit HRP labelled polymer for 30 min. Staining was completed by incubation with DAB substrate chromogen (DAKO) according to the manufacturer’s instructions.

### Statistical Analysis

Data are presented as box plots indicating median, lower and upper quartiles and range of the values. Statistical differences were analyzed by two-tailed Mann-Whitney U Test (SPSS Statistics 19.0 program, IBM, Leibniz Rechenzentrum, München, Germany) or Student’s t-test (MS Excel) and a value of p<0.05 was regarded as significant. The Pearson’s correlation was calculated using the IBM SPSS Statistics 19.0 program.

## Results

### MnSOD is Induced in Visceral Adipose Tissues of Rodents Fed a High Fat Diet

**Figure 1:**
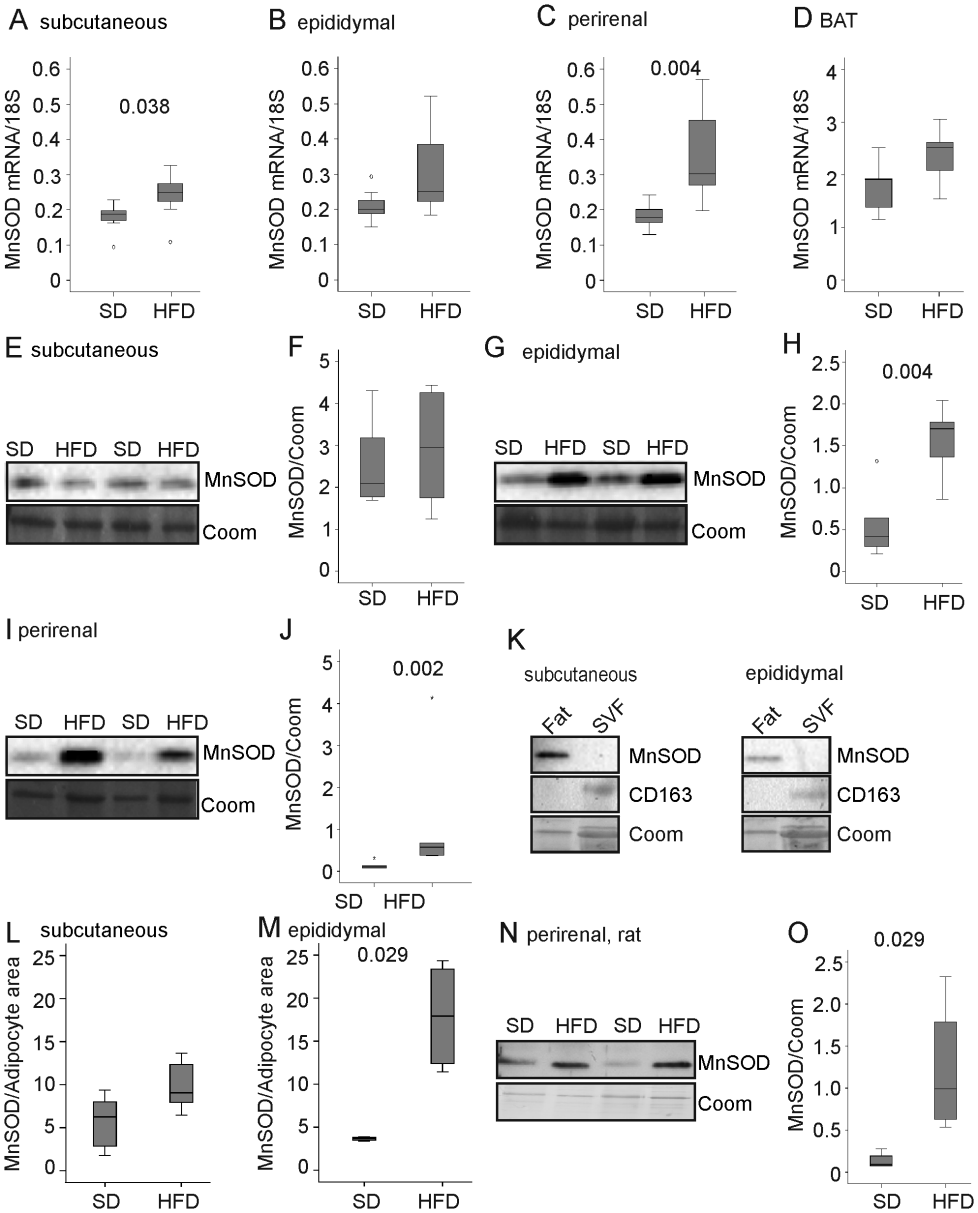
MnSOD in adipose tissues of male rodents fed high fat diets. MnSOD mRNA in subcutaneous (A), epididymal (B), perirenal (C) and brown adipose tissue (BAT, D) of mice fed a standard (SD) or a high fat diet (HFD). MnSOD protein in subcutaneous (E, F), epididymal (G, H), and perirenal (I, J) fat of mice fed a SD or HFD. Coomassie (Coom) stained gel is shown as loading control. (K) MnSOD and CD163 in subcutaneous and epididymal adipose tissue of mice fed a SD and in stromavascular cell fraction (SVF) isolated from these fat depots. (L) MnSOD protein/adipocyte area in subcutaneous (6 mice per group) adipose tissues of mice fed a SD or HFD. (M) MnSOD protein/adipocyte area in epididymal (4 mice per group) adipose tissues of mice fed a SD or HFD. (N) MnSOD protein in perirenal fat of rats fed a SD or a HFD. Coomassie (Coom) stained gel is shown as loading control. (O) Quantification of the data partly shown in N.

Expression of MnSOD mRNA was determined in subcutaneous, epididymal, perirenal and brown adipose tissue of mice kept on a standard chow (SD) or on a high fat diet (HFD). MnSOD mRNA was significantly higher in subcutaneous and perirenal fat and tended to be increased in epididymal fat (p = 0.097) and in brown adipose tissue (p = 0.053) of mice fed a HFD (Fig. 1A – D). Immunoblot analysis revealed strong induction of MnSOD in visceral fat depots while levels were not altered in subcutaneous fat of mice on a HFD (Fig. 1E–J).

Obesity is associated with a higher number of adipose tissue resident macrophages [29], and expression of the macrophage receptor F4/80 was significantly increased in all of these fat depots (Table S1). Correlation of F4/80 with MnSOD mRNA was r = 0.608 (p = 0.021) in subcutaneous fat, r = 0.817 (p<0.001) in epididymal fat, r = 0.805 (p = 0.001) in perirenal fat and r = 0.700 (p = 0.005) in brown adipose tissue. After correcting for body weight this correlation was only significant in brown fat (r = 0.636, p = 0.019).

MnSOD is expressed by most if not all cell types including adipocytes and macrophages [30]. Compared to total subcutaneous and epididymal fat MnSOD is, however, hardly detectable in the stromavascular cell fraction (SVF) isolated from the respective adipose tissues of mice fed a SD (Fig. 1K). CD163 which is specifically expressed by monocytes/macrophages [31] is more highly expressed in SVF (Fig. 1K). Quantification of MnSOD protein in subcutaneous and epididymal adipocytes using Image J software revealed increased MnSOD in epididymal adipocytes of mice fed a HFD when compared to cells of SD fed animals (Fig. 1L, M). Although this does not exclude that MnSOD is also altered in SVF these data clearly show that MnSOD is increased in epididymal adipocytes in obesity.

MnSOD protein was also significantly higher in the perirenal fat of Wistar rats kept on a HFD (Fig. 1N, O). MnSOD protein was comparably abundant in subcutaneous and perirenal fat of non-diabetic and diabetic Zucker rats with similar body weight (Table S2, 1.).

**Figure 2:**
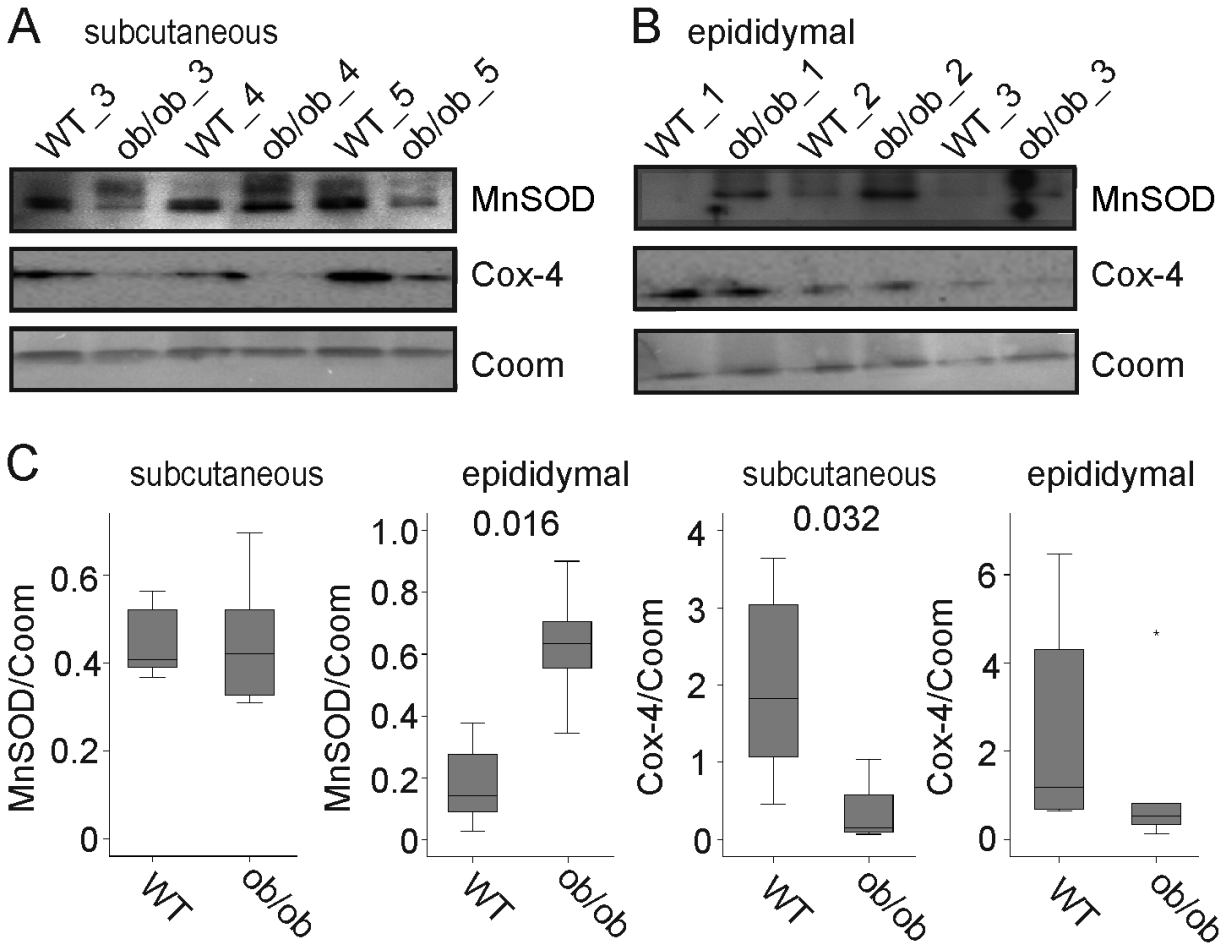
MnSOD in adipose tissue of ob/ob mice. (A) MnSOD and Cox-4 protein in subcutaneous fat of ob/ob mice and wild type (WT) animals. Coomassie (Coom) stained gel is shown as loading control. (B) MnSOD and Cox-4 protein in epididymal fat of ob/ob mice and wild type animals. Coomassie (Coom.) stained gel is shown as loading control. (C) Quantification of the immunoblots partly shown in A and B.

In epididymal fat of ob/ob mice MnSOD was also increased (Fig. 2B, C). In subcutaneous adipose tissue of ob/ob mice MnSOD protein was similar to control animals (Fig. 2A, C). Cytochrome C oxidase-4 (Cox-4) was significantly lower in subcutaneous fat of ob/ob mice (Fig. 2A, C) and was similarly expressed in epididymal fat of these mice (Fig. 2B, C). MnSOD to Cox-4 ratio was significantly higher in subcutaneous (p = 0.032) and epididymal adipose tissue (p = 0.016) of ob/ob mice (data not shown).

**Figure 3:**
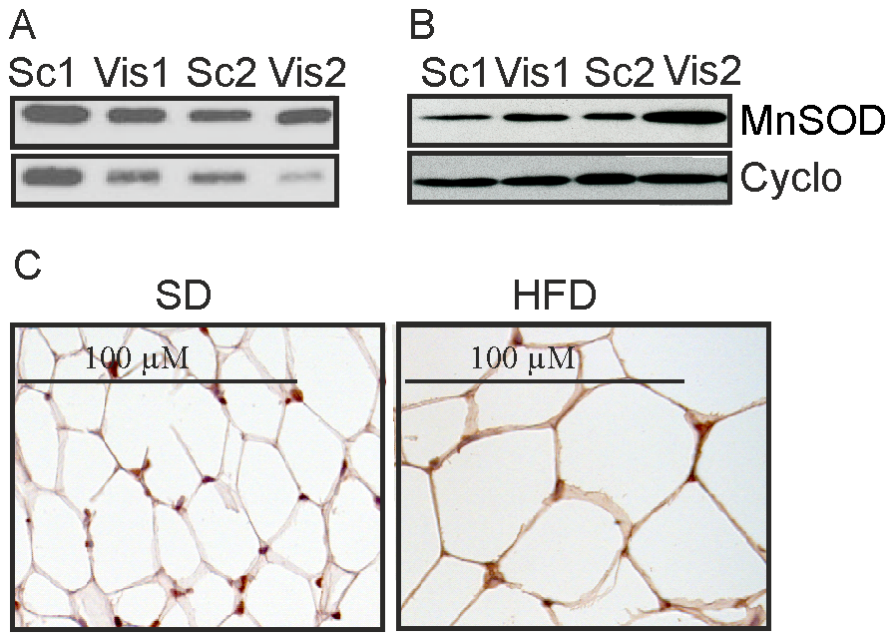
MnSOD in different adipose tissue depots. (A) MnSOD protein in subcutaneous (sc) and visceral (vis) fat of two mice. (B) MnSOD protein in paired samples of subcutaneous (sc) and visceral (vis) human adipocytes of two extremely obese patients. (C) Immunohistochemistry of MnSOD in subcutaneous fat of mice kept on a standard chow (SD) or high fat diet (HFD).

Brown adipose tissue has a high mitochondrial density [8] and MnSOD mRNA was about 10-fold more abundant when compared to white fat depots with similar MnSOD mRNA levels (Fig. 1A–D). MnSOD protein was equally abundant in subcutaneous and visceral fat of mice kept on a SD (Fig. 3A, Table S2, 2.). In paired samples of purified human subcutaneous and visceral adipocytes of two different morbidly obese patients MnSOD was 1.9 and 4.5 fold higher in visceral compared to subcutaneous fat (Fig. 3B). Immunohistochemistry demonstrated localization of MnSOD in adipocytes and adipose tissue resident macrophages in murine subcutaneous fat of 6 animals kept on a SD and 6 mice on a HFD (Fig. 3C and data not shown).

### Differentiation, Free Fatty Acids, IL-1α and Lipopolysaccharide Induce MnSOD

**Figure 4:**
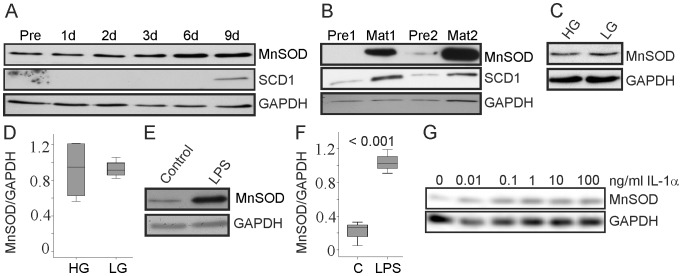
MnSOD in 3T3-L1 cells differentiated in the presence of glucose, LPS and IL-1α. (A) MnSOD and stearoyl CoA desaturase 1 (SCD1) protein in preadipocytes (Pre), 1 d, 2 d, 3 d, 6 d and 9 d differentiated cells. (B) MnSOD and SCD1 in primary human preadipocytes (Pre) and the respective mature cells (Mat) of two different donors. (C) MnSOD in 3T3-L1 cells differentiated in the presence of 25.0 mM (HG) and 5.5 mM (LG) glucose. (D) Quantification of the results of 4 experiments partly shown in C. (E) MnSOD in 3T3-L1 cells differentiated in the presence of LPS (10 ng/ml). (F) Quantification of the results of 3 experiments partly shown in E. (G) MnSOD in 3T3-L1 cells differentiated in the presence of increasing concentrations of IL-1α.

MnSOD was found upregulated during differentiation of 3T3-L1 cells [7] and this was confirmed in the present study (Fig. 4A, Table S2, 3.). Stearoyl CoA desaturase 1 (SCD1) is shown as control and was found induced in mature cells as described [32]. MnSOD and SCD1 protein were also higher in mature human subcutaneous adipocytes when compared to the respective preadipocytes (Fig. 4B, Table S2, 4.). Differentiation of 3T3-L1 cells in the presence of 5.5 mM instead of the usually used 25.0 mM glucose did neither affect lipid droplet formation (data not shown) nor MnSOD levels (Fig. 4C, D). Differentiation of 3T3-L1 cells in the presence of LPS (10 ng/ml) markedly induced MnSOD protein (Fig. 4E, F). Further, IL-1α which has been described to upregulate MnSOD in several cell lines [33] and is increased in serum of obese mice [34] induced MnSOD in 3T3-L1 cells (Fig. 4G, Table S2, 5.).

**Figure 5:**
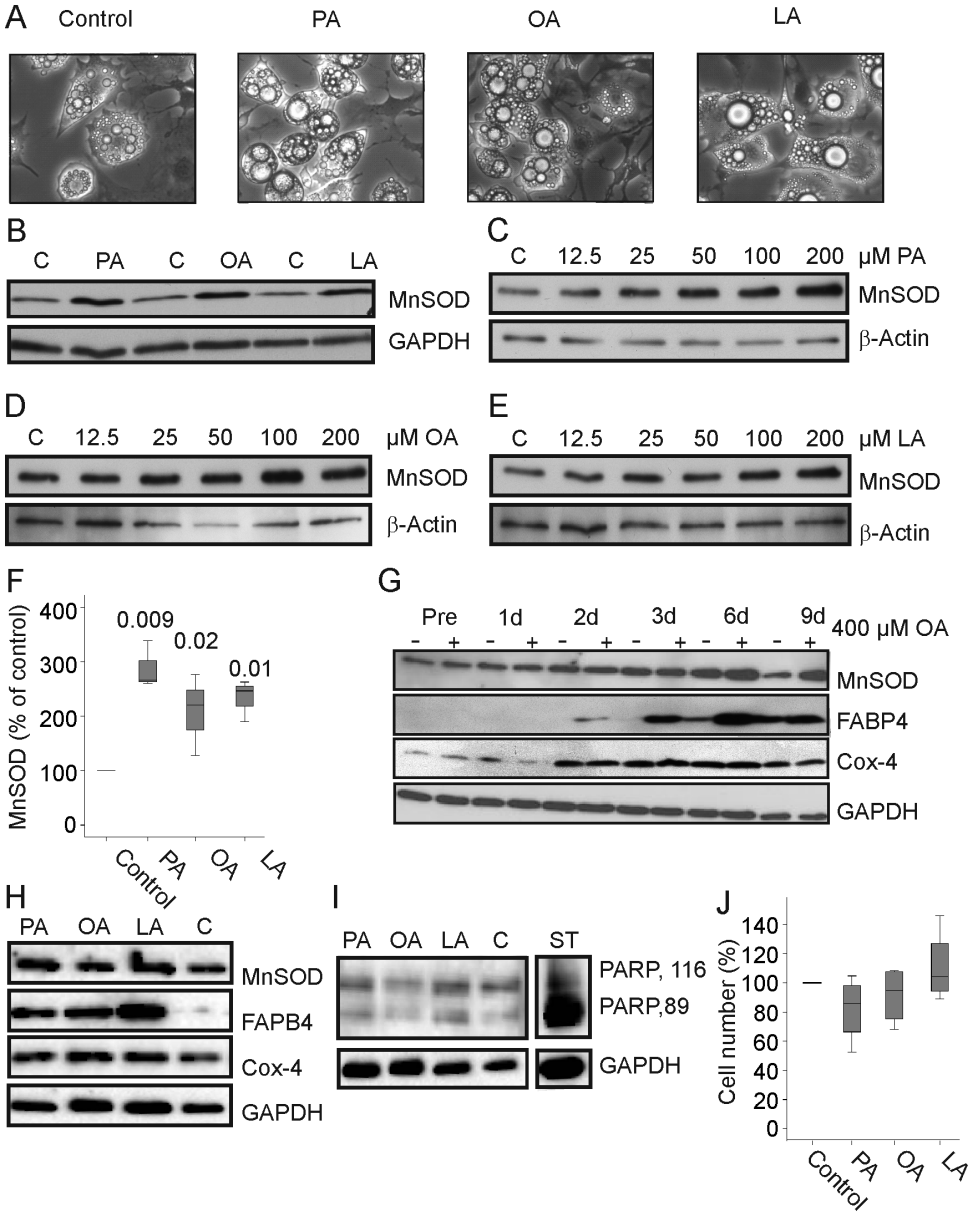
MnSOD in 3T3-L1 cells differentiated in the presence of free fatty acids. (A) Photomicrograph of 3T3-L1 cells differentiated with the standard protocol and in the presence of 200 µM palmitate (PA), oleate (OA) or linoleate (LA). (B) MnSOD in 3T3-L1 cells differentiated with the standard protocol (C) and in the presence of 200 µM PA, OA or LA. MnSOD in 3T3-L1 cells differentiated in the presence of increasing concentrations of PA (C), OA (D) or LA (E). (F) Quantification of the data of 3 PA incubated, 4 OA incubated and 3 LA incubated cells partly shown in B to E. (G) MnSOD, FABP4 and Cox-4 protein in preadipocytes (Pre) and cells differentiated for 1 d, 2 d, 3 d, 6 d and 9 d in the presence (+) or absence (−) of 400 µM oleic acid. (H) MnSOD, FABP4, Cox-4 and GAPDH in 3T3-L1 cells differentiated in the presence of 200 µM PA, OA or LA. (I) Full length (PARP, 116 kDa) and cleaved PARP (PARP, 89 kDa) in 3T3-L1 cells differentiated in the presence of 200 µM PA, OA or LA. ST indicates lysate of staurosporine treated 3T3-L1 cells as positive control which was analyzed on the identical gel in a non-adjacent lane. (J) Number of mature adipocytes when cells were differentiated in the presence of 200 µM PA, OA or LA. Data of 4 experiments are shown.

Differentiation of 3T3-L1 cells in the presence of 200 µM palmitate (PA), oleate (OA) or linoleate (LA) was associated with higher triglyceride levels (data not shown), formation of larger lipid droplets (Fig. 5A) and similarly raised MnSOD protein (Fig. 5B, F). Differentiation of 3T3-L1 cells with increasing concentrations of PA, OA and LA showed induction of MnSOD in cells treated with 50 µM, 100 µM and 200 µM PA, 100 µM and 200 µM OA and 100 µM and 200 µM LA (Fig. 5C – E). Immunoblot analysis of cell lysates obtained during differentiation revealed that upregulation of MnSOD by OA occurred during day 6 and day 9 (Fig. 5G). Fatty acid binding protein 4 (FABP4) was higher in the cells differentiated in the presence of OA as described [27], [35]. Cox-4 was found induced in early differentiation but was not affected by oleic acid (Fig. 5G, H). PA, OA and LA had no effect on Cox-4 whereas FABP4 and MnSOD were induced (Fig. 5H). Exposure of mature cells to PA, OA or LA for 24 h did not enhance MnSOD levels (data not shown) suggesting that upregulation of this protein is not a direct effect of increased lipid storage.

Poly ADP ribose polymerase (PARP) is cleaved by caspases in apoptotic cells [36] but distribution of full-length and cleaved PARP is not altered by differentiation of 3T3-L1 cells in the presence of FFA (Fig. 5I). The number of differentiated adipocytes was similar in cells differentiated in the presence or absence of FFA (Fig. 5J) indicating that cell death is not increased.

**Figure 6:**
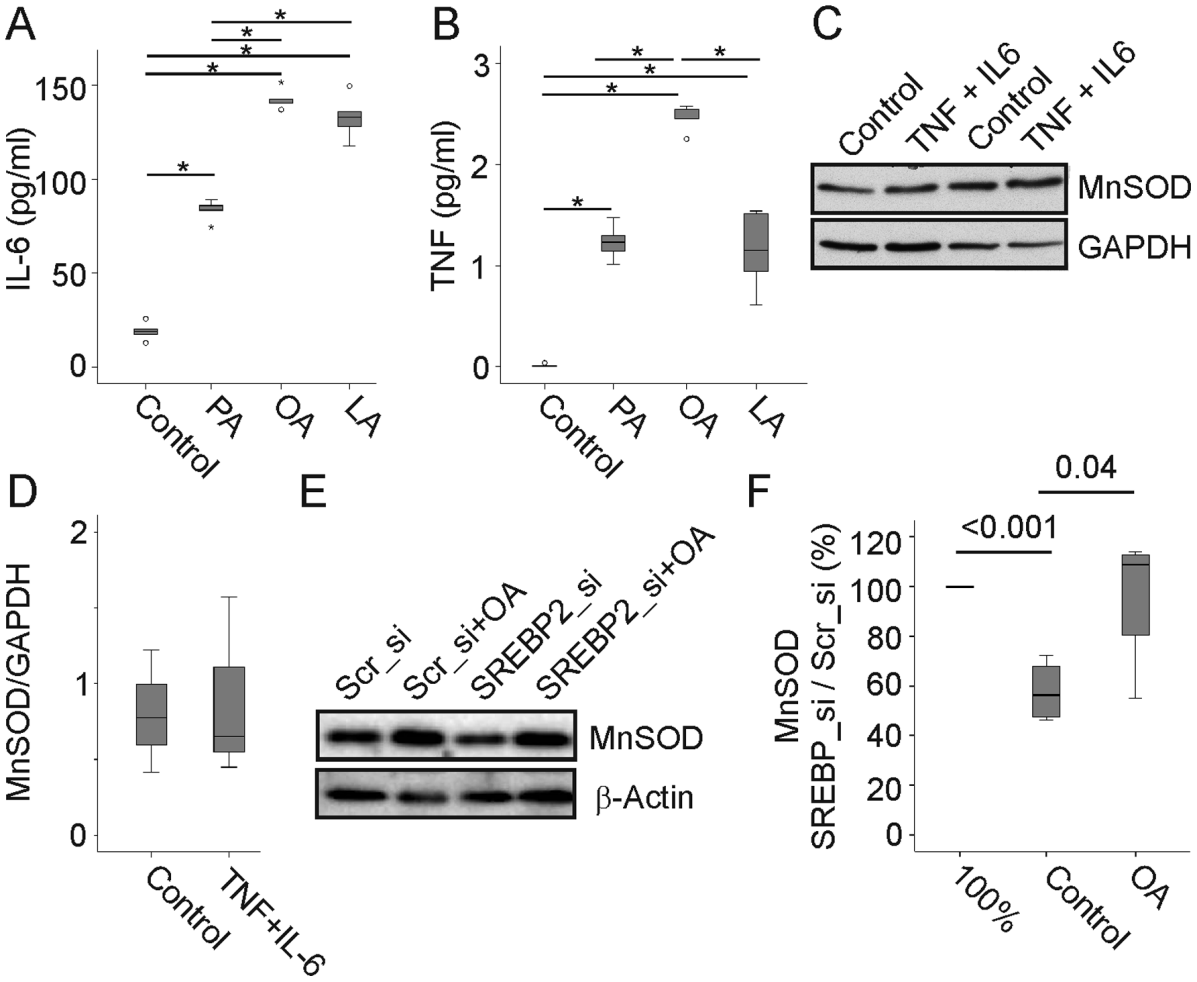
IL-6, TNF and SREBP2 knock-down in 3T3-L1 cells. (A) IL-6 in the supernatants of 3T3-L1 cells differentiated with the standard protocol and in the presence of 200 µM palmitate (PA), oleate (OA) or linoleate (LA) * is p-value <0.001. (B) TNF in the supernatants of 3T3-L1 cells differentiated with the standard protocol (C) and in the presence of 200 µM PA, OA or LA. * is p-value <0.001. (C) MnSOD in 3T3-L1 cells differentiated in the presence of TNF and IL-6. (D) Quantification of the data of 3 TNF/IL-6 incubated cells partly shown in C. (E) MnSOD and SREBP2 in adipocytes differentiated from preadipocytes treated with scrambled (Scr) or SREBP2 siRNA. Cells were differentiated with the standard protocol or in the presence of 200 µM OA. (F) Quantification of MnSOD in the cells described in E. Ratio of MnSOD protein in SREBP si RNA treated cells to scrambled siRNA transfected cells in % is shown. Data of 4 experiments have been calculated.

FFA exert proinflammatory effects and IL-6 and TNF were significantly induced by 200 µM PA, OA and LA as has already been shown for PA and OA [27] (Fig. 6A, B). Differentiation of 3T3-L1 cells in the presence of TNF (10 pg/ml), IL-6 (200 pg/ml) or both did, however, not affect MnSOD protein (Fig. 6C, D and data not shown). IL-1α which induces MnSOD (Fig. 4G) was measured in the supernatants of FFA treated 3T3-L1 cells. Concentrations were below the sensitivity (2.5 pg/ml) of the respective ELISA showing that 3T3-L1 cells release low if any IL-1α which most likely has no autocrine/paracrine effect on MnSOD.

Excess storage of triglycerides in hypertrophic adipocytes leads to a deficit of cellular cholesterol and activation of SREBP2 [27], [37]. Adipocytes differentiated from preadipocytes treated with SREBP2 siRNA have reduced cholesterol, triglycerides [27] and MnSOD (Fig. 6E, F). When differentiation of 3T3-L1 cells was performed in the presence of OA, cells transfected with scrambled or SREBP2 siRNA had similar levels of triglyceride (data not shown and [27]) and MnSOD (Fig. 6E, F).

### Knock-down of MnSOD does not Affect Triglyceride Storage of Predifferentiated and Mature Cells

**Figure 7:**
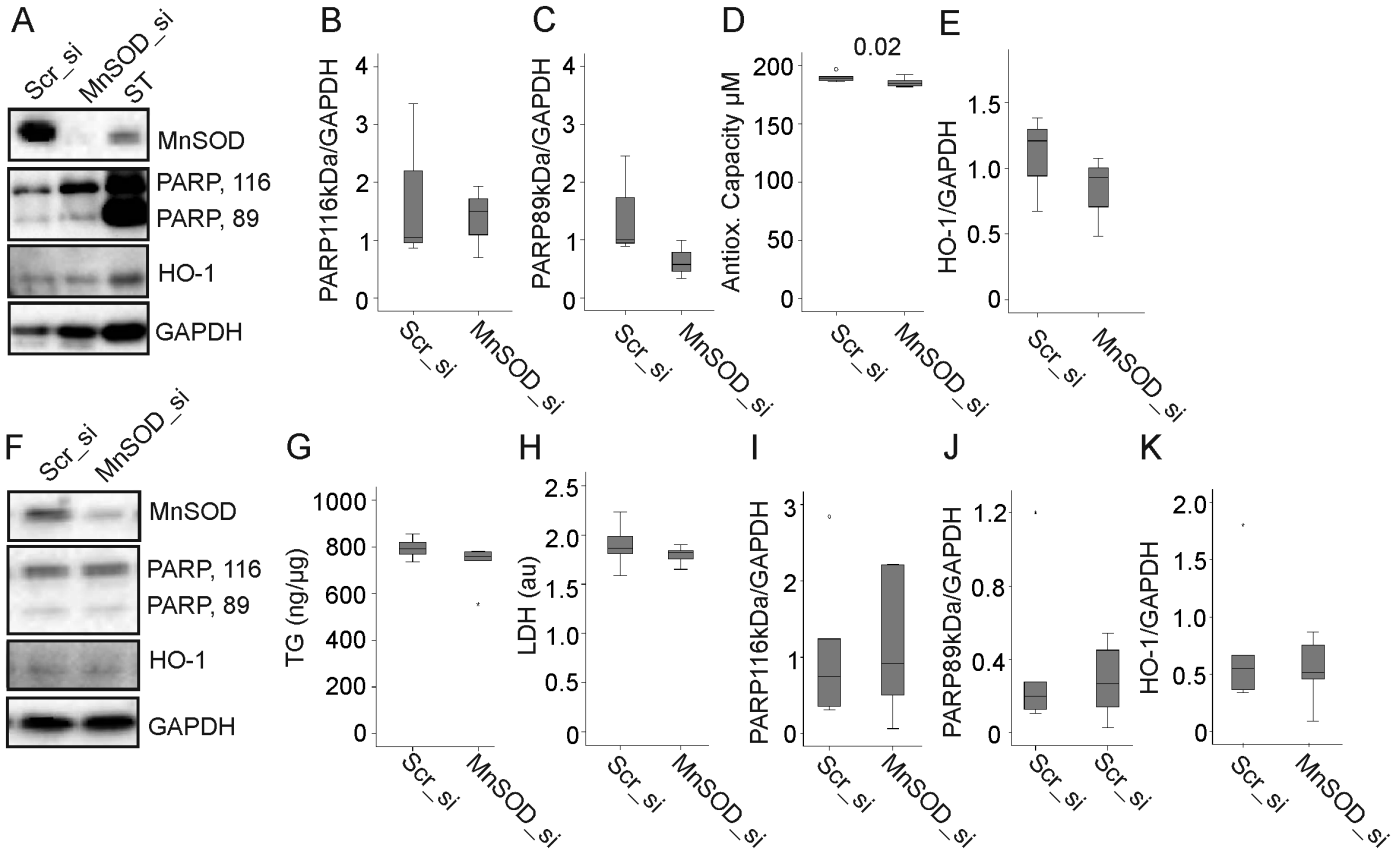
MnSOD knock-down in predifferentiated and differentiated 3T3-L1 cells. (A) MnSOD, PARP, HO-1 and GAPDH in mature 3T3-L1 cells treated with scrambled (Scr) or MnSOD siRNA. Staurosporine treated cells (ST) are shown as control. (B) Full-length PARP in scrambled (Scr) and MnSOD siRNA treated cells partly shown in A, data of 3 experiments have been calculated. (C) Cleaved PARP in scrambled (Scr) and MnSOD siRNA treated cells partly shown in A, data of 3 experiments have been calculated. (D) Antioxidative capacity of the cells described in A. Data of 6 experiments were calculated. (E) HO-1 in scrambled (Scr) and MnSOD siRNA treated cells partly shown in A, data of 3 experiments have been calculated. (F) MnSOD, PARP, HO-1 and GAPDH in 3T3-L1 cells differentiated from cells treated with siRNAs 6 d after initiation of differentiation. (G) Triglyceride levels (ng/µg cellular protein) in the cells described in F, data of 6 experiments were calculated. (H) Lactate dehydrogenase in the supernatants of cells described in F, data of 6 experiments have been calculated (arbitrary units = au). (I) Full-length PARP in scrambled (Scr) and MnSOD siRNA treated cells partly shown in F, data of 6 experiments have been calculated. (J) Cleaved PARP in scrambled (Scr) and MnSOD siRNA treated cells partly shown in F, data of 6 experiments have been calculated. (K) HO-1 in scrambled (Scr) and MnSOD siRNA treated cells partly shown in F, data of 6 experiments have been calculated.

Upregulation of MnSOD in FFA exposed 3T3-L1 cells led us to hypothesize that induction of this enzyme may protect the cells from harmful effects of lipid accumulation and thereby may facilitate triglyceride storage. Knock-down of MnSOD in fully differentiated 3T3-L1 cells markedly reduced MnSOD protein (Fig. 7A, Table S2, 6). MnSOD knock-down did not alter distribution of full-length and cleaved PARP (Fig. 7A–C). Antioxidative capacity of these cells was modestly but significantly reduced (Fig. 7D) while heme oxygenase-1 (HO-1) which is induced upon oxidative stress [38] was similar in control and MnSOD siRNA treated cells (Fig. 7A, E). Of note, triglyceride storage was not affected (data not shown). Cellular triglyceride storage was also not altered in 9 d matured 3T3-L1 cells where MnSOD was knocked down 6 d after initiation of differentiation (Fig. 7F, G), the time point where an upregulation of MnSOD was observed in the oleic acid incubated cells (Fig. 5G). MnSOD was significantly reduced in the mature adipocytes (Fig. 7F, Table S2, 7.) Cell viability was assessed by measuring lactate dehydrogenase in cell supernatants (Fig. 7H), counting number of differentiated adipocytes (data not shown) and analysis of PARP (Fig. 7F, I, J) and was not affected by MnSOD knock-down. HO-1 is not increased in the MnSOD siRNA treated cells (Fig. 7F, K).

## Discussion

In this study it is shown that MnSOD is strongly upregulated in visceral fat depots of obese rodents. This difference in not seen in fat depots of mice fed a standard chow showing that induction of this enzyme is associated with obesity. Compared with subcutaneous fat, visceral fat is characterized by a reduced capacity to store excess triglycerides, increased oxidative stress, higher infiltration with macrophages and inflammation [39], [40]. LPS and IL-1α, which are elevated in obesity [34], [41], upregulate MnSOD in 3T3-L1 cells. TNF is mainly released by adipose-tissue resident macrophages, and is further induced by FFA [42], [43], and also increases MnSOD in 3T3-L1 adipocytes [44]. Therefore, inflammation which is aggravated by FFA seems to contribute to elevated MnSOD in visceral fat depots in obesity. Expression of the macrophage membrane protein F4/80 positively correlates with MnSOD mRNA further indicating an association of MnSOD and inflammation. Number of adipose tissue resident macrophages is strongly increased in obesity and F4/80 is higher in all of the fat depots analyzed as has already been described [29], [45]. MnSOD protein is not raised in subcutaneous fat although F4/80 is increased suggesting that macrophages do not considerably contribute to total adipose tissue MnSOD protein. This suggestion is supported by the relatively low expression of MnSOD protein in SVF. Whether MnSOD is also induced in SVF in obesity has not been analyzed herein.

MnSOD is not regulated by glucose in 3T3-L1 cells and is not increased in adipose tissues of hyperglycemic Zucker diabetic rats excluding that high serum glucose contributes to raised MnSOD.

FFA are stored as triglycerides and excess fat accumulation impairs adipocyte function [39], [40]. Palmitate, oleate and linoleate in the concentrations used do not affect viability of 3T3-L1 cells, and palmitate in concentrations up to 750 µM has no considerable effect on apoptosis and necrosis of these cells [10]. Differentiation of 3T3-L1 cells in the presence of FFA increases triglycerides, release of proinflammatory cytokines [10], [27] and MnSOD. Current experiments exclude that TNF and IL-6 secreted by hypertrophic 3T3-L1 cells mediate MnSOD induction by an autocrine/paracrine effect. SREBP2 is activated in hypertrophic cells [27], [37] and adipocytes differentiated from 3T3-L1 fibroblasts with a knockdown of this transcription factor have reduced MnSOD. These cells store less triglycerides and levels are normalized by cultivation in the presence of OA [27]. MnSOD is no longer reduced in these cells suggesting that SREBP2 does not regulate MnSOD abundance. This experiment also demonstrates that it is quite difficult to identify the factors mediating upregulation of MnSOD by FFA during adipogenesis. Knock-down of specific genes in preadipocytes has multiple effects on subsequent adipogenesis [46]–[49] complicating the characterization of the specific pathways regulating induction of MnSOD.

In contrast to visceral fat, MnSOD protein is not induced in subcutaneous fat depots of obese animals. Analysis of MnSOD mRNA indicates a modest increase of its expression in mice on a HFD which is, however, not accompanied by higher protein levels. This is partly explained by the technique used to analyze MnSOD protein which is not appropriate to detect minor changes of protein expression. Posttranslational modifications also regulate MnSOD protein level [50] and this may explain why regulation of MnSOD mRNA is not in accordance with its protein expression.

About four weeks after HFD adipogenesis is initiated in epididymal fat while subcutaneous fat is enlarged by cell hypertrophy for at least 12 weeks [51]. Upregulation of MnSOD by FFA is only seen during differentiation suggesting that the different modes of fat tissue growth may also explain depot specific effects of MnSOD expression in obesity.

In subcutaneous adipose tissue of ob/ob mice Cox-4 is reduced and this may indicate a lower quantity of mitochondria as described [19]. MnSOD to Cox-4 ratio is increased in subcutaneous and visceral adipose tissues of ob/ob mice demonstrating that these proteins are not co-regulated. In line with this finding FFA raise MnSOD but not Cox-4.

Oxidative stress impairs adipocyte function and triglyceride storage [24], [52] and it was tested whether MnSOD has a role in lipid accumulation of 3T3-L1 cells. Knock-down of MnSOD in premature and mature adipocytes does not alter lipid storage capacity. Further, cell viability is not reduced. Heme oxygenase-1 which is induced by oxidative cellular stress [38] is not upregulated. Antioxidative capacity is only modestly lower when MnSOD is knocked-down implying a very little increase in ROS which may gradually lower antioxidative potential.

These results demonstrate that a strong decline of MnSOD in mature adipocytes is not associated with excess oxidative stress. Production of ROS has been determined in cells overexpressing MnSOD, in cells where this enzyme has been knocked-down or blocked by an inhibitor. In line with current findings ROS are not altered by any of these interventions as long as the cells are not challenged with agents causing oxidative stress [53]–[57]. Therefore, low levels of MnSOD are not associated with enhanced ROS in the basal state [53]–[57]. In situations of excessive ROS formation MnSOD exerts protective effects [53]–[57] and most likely advances oxidative stress of hypertrophic adipocytes.

During the preparation of this manuscript Pei-Wen et al. have published a paper showing higher MnSOD in visceral fat of mice fed a high sucrose high fat diet for 6 months [58] in accordance with our findings.

In summary current data suggest that inflammation and free fatty acids upregulate MnSOD in adipocytes, thus explaining the observed increase of MnSOD in visceral adipose tissues of obese rodents.

## Supporting Information

Table S1 **Expression of F4/80 mRNA in adipose tissues.** F4/80 mRNA in adipose tissues of mice fed a standard diet (SD) or a high fat diet (HFD), p-values are for comparison of F4/80 expression in the respective adipose tissues of SD and HFD fed animals.
(DOCX)

Table S2 **Quantification of immunoblot data.** Immunoblots were quantified using Image J and respective values, number of experiments/animals analyzed and p-values are given.
(DOCX)
