# Supplementary material for: Free Fatty Acids, Lipopolysaccharide and IL-1α Induce Adipocyte Manganese Superoxide Dismutase Which Is Increased in Visceral Adipose Tissues of Obese Rodents
Source: PLoS One. 2014 Jan 24;9(1):e86866. doi: 10.1371/journal.pone.0086866 (PMC3901719; doi:10.1371/journal.pone.0086866)
Supplement: Table S1 — Expression of F4/80 mRNA in adipose tissues. F4/80 mRNA in adipose tissues of mice fed a standard diet (SD) or a high fat diet (HFD), p-values are for comparison of F4/80 expression in the respective adipose tissues of SD and HFD fed animals. (DOCX) [file pone.0086866.s001.docx]

Table S1 Expression of F4/80 mRNA in adipose tissues

|  | **SD** | **HFD** | **p-value** |
| --- | --- | --- | --- |
| Subcutaneous fat | 0.32 (0.16 -0.36) | 0.43 (0.37 -0.47) | 0.001 |
| Epididymal fat | 0.29 (0.22 -0.34) | 0.64 (0.43 -2.05) | 0.001 |
| Perirenal fat | 0.20 (0.09 – 0.24) | 0.62 (0.36 -1.37) | 0.001 |
| Brown adipose tissue | 0.04 (0.03 – 0.06) | 0.08 (0.06 – 0.12) | 0.002 |
