# Supplementary material for: Free Fatty Acids, Lipopolysaccharide and IL-1α Induce Adipocyte Manganese Superoxide Dismutase Which Is Increased in Visceral Adipose Tissues of Obese Rodents
Source: PLoS One. 2014 Jan 24;9(1):e86866. doi: 10.1371/journal.pone.0086866 (PMC3901719; doi:10.1371/journal.pone.0086866)
Supplement: Table S2 — Quantification of immunoblot data. Immunoblots were quantified using Image J and respective values, number of experiments/animals analyzed and p-values are given. (DOCX) [file pone.0086866.s002.docx]

Table S2 Quantification of immunoblot data

1. MnSOD / cyclophilin in subcutaneous (sc) and perirenal adipose tissues of Zucker control rats (ZDL) and Zucker diabetic (ZDF) rats

| ZDL, sc | ZDF, sc | Number of animals per group | p-value |
| --- | --- | --- | --- |
| 1.21 (0.82-2.47) | 0.73 (0.50-0.99) | 4 | 0.116 |
|  |  |  |  |
| ZDL, perirenal | ZDF, perirenal |  | p-value |
| 1.88 (1.52-2.46) | 1.11 (0.30-2.53) | 4 | 0.280 |

2. MnSOD / cyclophilin in paired samples of subcutaneous (sc) and visceral (vis) adipose tissue of mice (Fig. 3A)

| sc | vis | Number of animals | p-value |
| --- | --- | --- | --- |
| 0.93 (0.66-1.27) | 1.4 (0.34-2.81) | 4 | 0.357 |

3. MnSOD / GAPDH in 3T3-L1 preadipocytes and in differentiated adipocytes (Fig. 4A)

| pre | mature | Number of experiments | p-value |
| --- | --- | --- | --- |
| 0.65 (0.56-0.83) | 1.00 (1.26-1.66) | 3 | 0.038 |

4. MnSOD / GAPDH in human preadipocytes and the respective differentiated adipocytes (Fig. 4B)

| pre | mature | Number of probands | p-value |
| --- | --- | --- | --- |
| 0.02 (0.01-0.03) | 0.52 (0.48-0.75) | 3 | 0.003 |

5. MnSOD / GAPDH (% of control) in mature 3T3-L1 cells differentiated in the presence of increasing concentrations of IL-1α. IL-1α concentrations are given in ng/ml (Fig. 4G). Data of two experiments (Exp. 1 and 2) are shown.

| IL-1α | 0 | 0.01 | 0.1 | 1 | 10 | 100 |
| --- | --- | --- | --- | --- | --- | --- |
| Exp. 1 | 100 | 381 | 400 | 423 | 422 | 557 |
| Exp. 2 | 100 | 373 | 350 | 471 | 609 | 589 |

6. MnSOD / GAPDH in mature 3T3-L1 cells transfected with MnSODsi RNA (Fig. 7A)

| Control siRNA | MnSOD siRNA | Number of experiments | p-value |
| --- | --- | --- | --- |
| 0.72 (0.70-1.48) | 0.02 (0.01-0.17) | 4 | 0.005 |

7. MnSOD / GAPDH in mature 3T3-L1 cells transfected with MnSODsi RNA at day 6 after initiation of differentiation (Fig. 7F)

| Control siRNA | MnSOD siRNA | Number of experiments | p-value |
| --- | --- | --- | --- |
| 1.52 (0.74-1.61) | 0.26 (0.12-0.42) | 6 | <0.001 |
